# Bovine gene polymorphisms related to fat deposition and meat tenderness

**DOI:** 10.1590/S1415-47572009000100011

**Published:** 2009-03-01

**Authors:** Marina R. S. Fortes, Rogério A. Curi, Luis Artur L. Chardulo, Antonio C. Silveira, Mayra E. O. D. Assumpção, José Antonio Visintin, Henrique N. de Oliveira

**Affiliations:** 1 Departamento de Reprodução Animal, Faculdade de Medicina Veterinária e Zootecnia, Universidade de São Paulo, São Paulo, SPBrazil; 2 Departamento de Melhoramento e Nutrição Animal, Faculdade de Medicina Veterinária e Zootecnia, Universidade Estadual Paulista Júlio de Mesquita Filho, Botucatu, SPBrazil; 3 Departamento de Química e Bioquímica, Instituto de Biociências, Universidade Estadual Paulista Júlio de Mesquita Filho, Botucatu, SPBrazil

**Keywords:** bovine gene polymorphisms, candidate gene, fat deposition, beef cattle

## Abstract

Leptin, thyroglobulin and diacylglycerol O-acyltransferase play important roles in fat metabolism. Fat deposition has an influence on meat quality and consumers' choice. The aim of this study was to determine allele and genotype frequencies of polymorphisms of the bovine genes, which encode leptin (*LEP*), thyroglobulin (*TG*) and diacylglycerol O-acyltransferase (*DGAT1*). A further objective was to establish the effects of these polymorphisms on meat characteristics. We genotyped 147 animals belonging to the Nelore (*Bos indicus*), Canchim (5/8 *Bos taurus* + 3/8 *Bos indicus*), Rubia Gallega X Nelore (1/2 *Bos tauru*s + 1/2 *Bos indicus*), Brangus Three-way cross (9/16 *Bos taurus* + 7/16 *Bos indicus*) and Braunvieh Three-way cross (3/4 *Bos taurus* + 1/4 *Bos indicus*) breeds. Backfat thickness, total lipids, marbling score, ribeye area and shear force were fitted, using the General Linear Model (GLM) procedure of the SAS software. The least square means of genotypes and genetic groups were compared using Tukey's test. Allele frequencies vary among the genetic groups, depending on *Bos indicus* versus *Bos taurus* influence. The *LEP* polymorphism segregates in pure *Bos indicus* Nelore animals, which is a new finding. The *T* allele of *TG* is fixed in Nelore, and *DGAT1* segregates in all groups, but the frequency of allele *A* is lower in Nelore animals. The results showed no association between the genotypes and traits studied, but a genetic group effect on these traits was found. So, the genetic background remains relevant for fat deposition and meat tenderness, but the gene markers developed for *Bos taurus* may be insufficient for *Bos indicus*.

## Introduction

Fat deposition demands beef industry attention for many reasons. To name a few, lean to fat deposition ratio improvement means better feed conversion efficiency, less husbandry cost and lower pressure on world feed supplies (Sillence, 2004). The marbling aspect associates with intramuscular fat deposition, interfering with consumer habits and meat pricing (Killinger *et al.*, 2004). Controlling backfat deposition is important because carcass quality and backfat thickness measurements are strongly associated with percentage of retail product. The weight of retail cuts, a trait of economic importance, is related to the ribeye area (Tait *et al.*, 2005). Last but not least, fat deposition, especially intramuscular fat, can interfere with meat tenderness perception (Crouse *et al.*, 1989).

The *LEP* gene encodes leptin, a 16-kDa protein produced by adipocytes and implicated in food intake regulation, energy balance, reproduction efficiency and fat deposition (Houseknecht *et al.*, 1998). Additionally, serum leptin has been correlated with fat deposition traits in cattle (Geary *et al.*, 2003), and *LEP* is likely associated to the *BM1500* microsatellite, which is implicated in fat content of beef carcasses (Fitzsimmons *et al.*, 1998). After Stone *et al.* (1996) mapped *LEP* to chromosome 4, many polymorphisms were described and associated with countless traits, from carcass fat content to fertility (Pomp *et al.*, 1997; Buchanan *et al.*, 2002; Barendse *et al.*, 2005; Schenkel *et al.*, 2005; Van Der Lende *et al.*, 2005).

The *TG* gene encodes thyroglobulin, the precursor of triiodothyronine and tetraiodothyronine, signals for fat cells development (Ailhaud *et al.*, 1992; Darimont *et al.*, 1993). Subcutaneous fat thickness and fat percentage of tissues in general, including milk, are expected to be influenced by *TG* polymorphisms because the iodothyronines affect adipocyte differentiation and the thyroid hormone levels influence milk fat percentage (Folley and Malpress, 1948). Attempts to associate *TG* markers and marbling or other fat deposition traits have been previously made, and *TG* polymorphisms are included in commercial panels (Barendse, 1999; Barendse *et al.*, 2004; Casas *et al.*, 2005; Rincker *et al.*, 2006; Van Eenennaam *et al.*, 2007).

Many association studies support the diacylglycerol O-acyltransferase 1 gene (*DGTA1*) as a marker for fat deposition traits (Thaller *et al.*, 2003; Kühn *et al.*, 2004; Tantia *et al.*, 2006). *DGTA1* encodes the catalyst enzyme of the reaction between diacylglycerol and acyl-CoA. This reaction is a final step in the synthesis of triglyceride, a major fat component. So, the enzyme encoded by *DGTA1* regulates the rate of triglycerides in adipocytes (Coleman and Bell, 1976) and has been implicated in energy homeostasis (Havel, 2001). In addition, *DGTA1* maps to chromosome 14, where *TG* is located, which is bodily related to the DNA marbling score marker CSSM66 (Barendse *et al.*, 1997).

In spite of the support from the literature for selecting the candidate genes mentioned above, some conflicting reports show no association of these genes with fat deposition traits (Rincker *et al.*, 2006). Also, most studies were conducted with *Bos taurus* cattle. Thus, the segregation and the predictive value of the *LEP*, *TG*, *and DGAT1* polymorphisms for fat deposition and meat quality traits were yet to be analyzed in *Bos indicus* (Nelore breed and Nelore crossbreed cattle).

The objectives of the present study were to estimate the allele and genotype frequencies of single nucleotide polymorphisms (SNP) of the *LEP*, *TG* and *DGAT1* genes and to associate genotypes with backfat thickness, total lipids (objective intramuscular fat deposition), marbling (subjective intramuscular fat deposition), ribeye area and shear force. The polymorphisms selected for the study were *LEP/Kpn*2I (Buchanan *et al.*, 2002), *TG/Psu*I (Thaller *et al.*, 2003) and *DGAT1/Cfr*I (Winter *et al.*, 2002).

## Material and Methods

### Animals

The study included carcasses of Nelore breed and of crosses that use Nelore as a formation breed, consisting on five different genetic groups. The genetic groups were classified as follows: 46 animals were Nelore - N - (*Bos indicus*), 41 Canchim - C - (5/8 *Bos taurus* + 3/8 *Bos indicus*), 19 Brangus Three-way cross - B3x - (9/16 *Bos taurus* + 7/16 *Bos indicus*), and 15 Braunvieh Three-way cross - BV3x - (3/4 *Bos taurus* + 1/4 *Bos indicus*). These animals were from the experimental feedlot facility of the *Department of Genetics and Animal Nutrition - FMVZ, Botucatu, SP, Brazil*. These cattle were sampled from four different farms, and the production system used in the university facility has been previously described in detail (Curi *et al.*, 2005). Additional 26 Rubia Gallega X Nellore crossbred - RGxN - (1/2 *Bos taurus* + 1/2 *Bos indicus*) were sampled from a semi-intensive system. All animals were slaughtered within the age gap of 15 to 19 months.

After humanitarian slaughter, performed in a supportive abattoir, carcasses were identified and cooled for 24 h, following which 2.50 cm-thick samples/steaks were removed from the *longissimus dorsi* muscle, between the 12^th^ and the 13^th^ ribs. The ribeye area (REA), also referred to as *longissimus dorsi* area, was measured at the abattoir by the quadrant methodology described in the USDA Quality Grade (USDA, 1989). The steaks were vacuum-packaged and aged at 1 to 2 °C until 14 days *postmortem* and then frozen until the further analyses were carried out.

### Fat deposition and meat quality traits

Further phenotypic analyses such as backfat thickness (BT), total lipids (TL), marbling score (MS), and shear force (SF) measurements were performed at the Chemistry and Biochemistry Department of the Institute of Biosciences of the São Paulo State University (UNESP, Botucatu, SP, Brazil), as described below.

Backfat thickness was measured with a caliper, following the methodology described in the USDA Quality Grade (USDA,1989). Marbling score was visually assessed (subjective scores from 1 to 5), according to the methodology described by the Aus-Meat Ltd *(*2001). Total lipids were evaluated using the method of Bligh and Dyer (1959). In short, six to eight cores of one inch x inch of meat were removed from the central area of each *longissimus dorsi* steak, carefully avoiding visible fat tissue. As mentioned above, the steaks used for this sampling were removed from the area between the 12^th^ and the 13^th^ ribs and were 2.54 cm thick. Then, the cores were minced and homogenized, before weighing the 5 g used for lipids assessment. Chloroform and methanol were added to the 5 g samples and rocked for over 30 min, for lipid extraction. Then, the samples were centrifuged to separate three phases: hydrophilic (disposed), solid (disposed), and hydrophobic solution (used for volumetric measurements). Five milliliters of the hydrophobic phase were transferred to a previously weighed 50 mL beaker flask and let to dry overnight. The lipid content was calculated by the weight difference of the beaker. The lipid assessment of each animal was done in duplicate, and the final value is the mean of both results. Shear force was determined according to Wheeler *et al.* (1997), as follows. The frozen meat samples were thawed under refrigerated conditions (4 °C for as long as 24 h) until reaching an internal temperature of 5 to 6 °C, cooked until reaching an internal temperature of 71 °C, cooled during 24 h at 5 to 6 °C, and then cylindrical pieces of 1.27 cm in diameter were removed from each sample, parallel to muscle fiber orientation. The cores were cut in a Warner-Bratzler shear force measurement equipment (speed of 20 cm/min, with a 25 kg capacity).

### DNA extraction and genotyping

After powdering meat samples with liquid nitrogen, DNA extraction was performed by digestion with protease K and precipitation with NaCl and alcohol, a non-phenolic method (Sambrook *et al.*, 1989). After extraction, each DNA solution was checked for quantity and integrity by agarose gel electrophoresis, then diluted to work concentration (10 ng/μ) and stored at -20 °C until genotyping.

The animals were genotyped for the *LEP, TG* and *DGAT1* genes by using the polymerase chain reaction - restriction length polymorphism (PCR-RFLP) technique. Alleles *C* and *T* of the *LEP* gene were determined by the amplification of a 94 bp fragment in exon 2, followed by digestion with *Kpn*2I, as reported by Buchanan *et al.* (2002). For the determination of alleles *C* and *T* of the *TG* gene, a 548 bp fragment located at the 5' untranslated region was amplified and digested with the *Psu*I restriction enzyme, as described by Thaller *et al.* (2003). Alleles *A* and *K* of the *DGAT1* gene were identified by the amplification of a 411 bp sequence corresponding to a fragment of exon 8, followed by digestion with *Cfr*I, as reported by Lacorte *et al.* (2006).

After digestion of the amplified *LEP, TG* and *DGAT1* gene products, the DNA fragments were separated, respectively, on 3.5, 2 and 2% agarose gels in a horizontal electrophoresis system. A standard molecular weight of 100 bp was applied onto each gel, next to the amplified and digested DNA fragments, so their size could be estimated. Ethidium bromide staining and exposure to ultraviolet light were used to visualize the DNA fragments in the gel. Using a digital photo-documentation system, the gels were photographed for ulterior data analyses. Individual genotypes were determined for each polymorphism by analyzing the size (in bp) of the fragments.

### Statistical analysis

Genotype and allele frequencies were calculated for each polymorphism according to Weir (1990). Differences in allele frequencies of the polymorphisms within and between genetic groups were determined by the method of Goodman adapted by Curi and Moraes (1981).

The traits of interest were analyzed by least square analysis of variance (p = 0.05), using the General Linear Model (GLM) procedure of the SAS program (SAS Institute Inc, 2004). The linear model used to fit the quantitative variables included, in addition to the genotype effect, the interaction between genetic groups and contemporary groups, as follows: *Yijk* = μ + G*i* + GGC*j* + e*ijk*, where *Yijk* = production trait, μ = overall mean, G*i* = fixed effect of the *i*^th^ genotype (*i* = 1,.., 3), GGC*j* = fixed effect of the *j*^th^ genetic group and contemporary group combined (*j* = 1,..., 9), and *eijk* = random error. The criteria for the contemporary groups included variations of sex, age at slaughter, feedlot and farm of origin. Animals with the same age, feedlot and farm of origin were slaughtered on the same day. In other words, the interaction between genetic and contemporary groups resulted in 9 subgroups, as follow: Canchim FE, Canchim MF, Nelore MA, Nelore MB, Nelore MD, Rubia Galega x Nelore FC, Rubia Galega x Nelore MC, Brangus Three-way cross, and Braunvieh Three-way cross MF. In these subgroups, the first letter represents the sex of the animals (F = female, M = male) and the second letter designates the day of slaughter (A,..., F). The least square means for genotypes and genetic subgroups were established and compared using the Tukey test. Genotypes with very low frequency (less than 0.10) in the total sample of individuals or genetic groups showing a single genotype were not included in the analysis, in order to prevent unreliable results. For the same reason, when most of the animals (over 80%) of one genotype belonged to the same genetic group, this genotype was entirely excluded from the analyses. The sire effect was not included in the linear model because the number of genotyped animals which were progenies of the same sire was very small. So, the possibility of confounding the influence of the genotype effect and of the sire effect on production traits was low because of the large number of small half-sib families.

## Results

The allele and genotype frequencies of all three polymorphisms in each genetic group and in the sample as a whole are summarized in Table 1 and 2, respectively.

The two allelic forms of *LEP* (*C* and *T*) were observed in all five genetic groups. Two restriction fragments of 75 and 19 bp, respectively, were seen when the *CC* genotype was present, and the *TT* genotype was reported from the observation of a 94 bp fragment. Heterozygotes presented all three fragment sizes: 94, 75 and 19 bp. The genetic groups with higher *Bos indicus* influence (Nelore and Rubia Gallega X Nelore) presented a much lower frequency of allele *T* (4.3 and 7.7%, respectively) and no animal with genotype *TT*. The two Three-way crosses presented similar *T* frequencies (20 to 26.3%) and were different from the Canchim breed (39%).

For *TG*, two allelic variants were reported: *C* (295 and 178 bp restriction fragments) and *T* (intact PCR product 473 bp fragment). *TG* alleles *C* and *T* were segregating in most genetic groups, but allele *C* was fixed in the Nelore animals, resulting in the occurrence of only the *CC* genotype. Rubia Gallega X Nelore presented a very low frequency of *T* (1.9%), with no statistically significant difference from the segregation of this polymorphism in the pure Nelore group. In fact, the occurrence of allele *T* increased numerically with the higher *Bos taurus* influence on each genetic group: Brangus Three-way cross (15.8%), Canchim (22%), and Braunvieh Three-way cross (33.3%).

The *DGAT1* alleles (*K* and *A*) segregated in all genetic groups*.* When only allele *K* was present, the fragment identified was like the intact PCR product, with 411 pb in size. When only the alanine-bearing allele was present, two fragments were observed (208 and 203 pb, respectively). Heterozygous individuals were determined by the presence of fragments of three sizes: 411, 208 and 203 bp, respectively. In contrast, allele *A* was less frequent in the Nelore breed, with a presence of only 5.4%, compared to a range of 53 to 67% in the other four genetic groups.

The least square means and standard errors of quantitative meat traits for the different subgroups are shown in Table 3. A subgroup effect was found for all meat traits: backfat thickness (p = 0.0022), total lipids (p = 0.0002), ribeye area (p < 0.0001), and shear force (p < 0.0001).

The least square means and standard errors of quantitative meat traits for the different genotypes of polymorphisms *DGAT1/Cfr*l *TG/Psu*I and *LEP/Kpn*2I are shown in Table 4. Genotypes *CC* and *CT* were analyzed for an association with *LEP/Kpn*2I, but no significance was found for any of the traits: backfat thickness (p = 0.1038), total lipids (p = 0.6298), ribeye area (p = 0.3355) and shear force (p = 0.9189). To characterize the effects of *TG/Psu*I, genotypes *CC* and *CT* were tested. No effect was found for *TG/Psu*I on backfat thickness (p = 0.7101), total lipids (p = 0.2813), ribeye area (p = 0.8044) or shear force (p = 0.4361). For the analysis of polymorphism *DGAT1/Cfr*l, only genotypes *AA* and *AK* were considered, and no significant effect was found regarding an association with backfat thickness (p = 0.5244), total lipids (p = 0.6293), ribeye area (p = 0.8235) or shear force (p = 0.2124).

The visual assessment of marbling revealed to be ineffective for the Nelore or *Bos indicus* crosses subgroups studied because of its very low variability, shown by the fact that most animals scored 1 and only few individuals scored 2. So, no association tests were made for this trait.

## Discussion

In 1997, Pomp *et al.* (1997) found an RFLP marker in the *LEP* gene using the enzyme *Sau*3AI that segregates in numerous *Bos taurus* breeds but was fixed in Brahman cattle (*Bos indicus*), which made segregation and association studies in this case impossible. Buchanan *et al.* (2002) used the enzyme *Kpn*2I to assess a substitution of cytosine (*C*) for thymine (*T*) at exon 2 of the *LEP* gene (AF120500)*,* assumed to cause an arginine to cysteine exchange, in Angus Hereford and Charolais breeds (*Bos taurus*). The overall allele frequency for the *LEP* gene polymorphism reported by these authors was 54% (*C*) and 46% (*T*), figures that seemed different from the 81% (*C*) and 19% (*T*) frequencies found in the present study. A difference between *Bos taurus* animals and *Bos indicus* or *Bos indicus* crosses was actually expected. In fact, even within *Bos taurus* cattle the allele frequencies may be distinct in British (higher *T* frequency) and in continental breeds (higher *C* occurrence) (Buchanan *et al.*, 2002).

Leptin polymorphisms have been associated with many characteristics of economic importance for livestock, including feed intake, milk yield, and carcass traits (Van der Lende *et al.*, 2005). A study on *Bos taurus* (Angus, Charolais, Limousin and Simmental) found an association between two leptin exon 2 polymorphisms and lean yield (Schenkel *et al.*, 2005). In *Bos taurus* animals, average fat and grade fat were shown to be affected by the genotype, and animals homozygous for allele *T* produced more leptin mRNA than those homozygous for allele *C* (Buchanan *et al.*, 2002), but no similar correlations with other fat deposition traits (backfat thickness and total lipids) were found in *Bos indicus* and *Bos indicus* crosses (present data). The results presented here are consistent with the association study that genotyped 3129 individuals, including many *Bos taurus* breeds and 317 *Bos indicus* (Brahman) animals, conducted by Barendse *et al.* (2005), who found no association between *LEP/Kpn*2I and marbling, backfat thickness, intramuscular fat and adjusted total fat. In the present study, no correlation was found for REA and *LEP*, although Geary *et al.* (2003) found a negative correlation between the *longissimusdorsi* area and serum leptin, and moreover the serum concentrations of leptin were significantly associated with carcass composition (marbling, backfat thickness and kidney, pelvic and heart fat) and quality grade, in crossbred *Bos taurus* (1/2 Angus + 1/4 Charolais + 1/4 Tarentaise).

Polymorphism at the 5' untranslated region of the thyroglobulin *TG* gene was patented by Barendse (1999) and is evaluated using *Psu*I to distinguish alleles *C* and *T* (Thaller *et al.*, 2003). The results obtained in the present study show allele *T* to be less frequent than *C*. Similar figures - 22 to 25% *T* frequency - have been reported before for *Bos taurus* breeds (Thaller *et al.*, 2003; Moore *et al.*, 2003), and it is likely that the ranging for Canchim and both Three-way crosses is a little wider (15 to 33%), due to the genetic influence of Nelore. A low *T* frequency was expected for *Bos indicus* cattle (Casas *et al.*, 2005), but this is the first record in Nelore, in which *C* is fixed. Many previous studies have acknowledged *TG/Psu*I effects on: marbling (Barendse, 1999), backfat thickness and ribeye area (Casas *et al.*, 2005), intramuscular fat (Thaller *et al.*, 2003), and percent of retail cuts and carcass weight EPDs (Rincker *et al.*, 2006). Moreover, Barendse *et al.* (2004) suggested that *TG/Psu*I is a causative mutation within the marbling QTL and that *T* is a favorable allele to intramuscular fat deposition. Yet, conflicting findings have been reported, and our results corroborate those which did not find any association between *TG* polymorphism and marbling or tenderness score in *Bos indicus* Brahman cattle (Casas *et al.*, 2005) or marbling, intramuscular fat, ribeye area and fat thickness in *Bos taurus* Simmental steers (Rincker *et al.*, 2006), or even backfat EBV in *Bos taurus* (Moore *et al.*, 2003). Thyroglobulin *C* to *T* variation is analyzed in the commercially available panel GeneSTAR Quality Grade (Genetic Solutions/Bovigen Pty. Ltd.), and the latest validation study confirmed the presence of allele *T* in an increasing number of carcasses graded Choice or Prime, although marbling was not associated with the marker (Van Eenennaam *et al.*, 2007). In the present study, the low frequency of the favorable allele made it difficult to establish an association of the polymorphism with carcass traits because of the small number of phenotypic data recorded for the *TT* genotype (only 4 animals).

Winter *et al.* (2002) discovered the *DGAT1/Cfr*I polymorphism, which is a nonconservative substitution of two *GC* nucleotides by *AA* at positions 10433 and 10434 (AJ318490). This substitution imposes a protein substitution of lysine by alanine (*K232A*), and these authors associated the lysine allele with higher milk fat content, suggesting the polymorphism as a causative mutation of the QTL for milk fat content. This original study observed that the *K* variant was more common in Jersey animals (about 80% frequency), Holstein-Friesian and Anatolian Black animals (about 35% frequency) than in other *Bos taurus* beef breeds (less than 20% of the lysine form). Previous studies on *Bos indicus* beef breeds found them to have the *K* allele fixed (Winter *et al.*, 2002; Tantia *et al.*, 2006) or at very high frequency (Casas *et al.*, 2005). Lacorte *et al.* (2006) reported that allele *K* was fixed in Brazilian *Bos indicus* breeds (Nelore and Guzerat). The novelty presented by our findings was the low frequency (5.4%) revealed for the occurrence of allele *A* in pure Nelore animals. In this study, crossbreeds (*Bos indicus* X *Bos taurus*) presented a 33 to 48% frequency of allele *K*, a value that is intermediary between the figures found in the literature (Winter *et al.*, 2002; Moore *et al.*, 2003) for *Bos taurus* beef breeds and the results found in this study for *Bos indicus*. The present findings, namely the lack of association between *K* and higher marbling or higher total lipids, are not consistent with literature statements that consider *DGAT1/Cfr*I to be the causative polymorphism for fat deposition traits (Grisart *et al.*, 2004). However, a discrepancy about the effects of *DGAT1* in cattle with different genetic backgrounds is already known, once it is significant for intramuscular fat content in German Holstein but not in Charolais animals (Thaller *et al.*, 2003). Our results are in agreement with those of Casas *et al.* (2005), who found no association between *K232A* alleles and backfat thickness, marbling, tenderness score or ribeye area in Brahman (*Bos indicus*) cattle. Kühn *et al.* (2004) reported a variable number of tandem repeats (VNTR) located upstream of *DGAT1*, which has an effect on milk fat content even among *AA* individuals, further clarifying the BTA 14 QTL. It is possible that 5' VNTR *DGAT1* contributes to fat deposition variability in *Bos indicus* animals.

According to Crouse *et al.* (1989), higher scores for tenderness are achieved in a sensory panel when intramuscular fat content is over 5%. Previous reports showed association of *LEP* with tenderness (Schenkel *et al.*, 2005). Thus, the effect of *LEP, TG* and *DGAT1* gene polymorphisms on shear force (mechanical evaluation of tenderness) was tested, but no association was found. Discrepancies among association studies may be related to different genetic backgrounds and variable epistatic effects and/or to environmental and management pressure on phenotypic data (Dekkers, 2004). The combined genetic-environmental component, here represented by the subgroups, has an influence on all analyzed traits. Furthermore, comparing the results obtained for the subgroups for each analyzed trait, it seems that subgroups within the same breed type tend to share similar results. In contrast, subgroups from distinct breed types presented distinct results.

Hence, while the genetic (or breed type) effect on meat traits continues to be of importance, the gene markers, which underpin this effect, are yet to be discovered for *Bos indicus*-influenced cattle. In other words, the fixation (or very low frequency) of alleles in *Bos indicus* (Nelore) and the lack of additive value shown by the present results for these markers at *LEP, TG* and *DGAT1* encourage a search for new markers. Adequate new marker panels should be developed specifically for *Bos indicus* cattle, to allow marker-assisted selection to be successful in Brazil.

## Figures and Tables

**Table 1:** Allele frequencies of *LEP, TG* and *DGTA1* polymorphisms in five bovine genetic groups and in the sample as a whole.

Polymorphism	Allele	Genetic group					Total
		N (n = 46)	RGxN (n = 26)	C (N = 41)	B3x (n = 19)	BV3x (n = 15)	
*LEP/Kpn*2I	*C*	0.957^A;a^	0.923^A;a^	0.610^B;ab^	0.737^AB;a^	0.800^AB;a^	0.810
	*T*	0.043^B;b^	0.077^B;b^	0.390^A;ab^	0.263^AB;b^	0.200^AB;b^	0.190

*TG/Psu*I	*C*	1.000^A;a^	0.981^A;a^	0.780^B;a^	0.842^AB;a^	0.667^B;ab^	0.881
	*T*	0.000^B;b^	0.019^B;b^	0.220^A;b^	0.158^AB;b^	0.333^A;ab^	0.119

*DGAT1/Cfr*l	*K*	0.946^A;a^	0.462^B;ab^	0.329^B;b^	0.474^B;ab^	0.367^B;ab^	0.568
	*A*	0.054^B;b^	0.538^A;ab^	0.671^A;a^	0.526^A;ab^	0.633^A;ab^	0.432

N = Nelore; RGxN = Rubia Gallega X Nelore; C = Canchim; B3x = Brangus Three-way cross; BV3x = Braunvieh Three-way cross. ^A, B^ = Different allele frequencies between genetic groups (p < 0.05). ^a, b =^ Different allele frequencies within genetic groups (p < 0.05).

**Table 2:** Genotype frequencies of the *LEP, TG* and *DGTA1* gene polymorphisms obtained for five genetic groups and in the sample as a whole.

Polymorphism	Genotype	Genetic group					Total
		N (n = 46)	RGxN (n = 26)	C (N = 41)	B3x (n = 19)	BV3x (N = 15)	
*LEP/Kpn*2I	*CC*	0.913	0.846	0.415	0.526	0.667	0.687
	*CT*	0.087	0.154	0.390	0.421	0.267	0.245
	*TT*	0.000	0.000	0.195	0.053	0.067	0.068

*TG/Psu*I	*CC*	1.000	0.962	0.610	0.648	0.467	0.789
	*CT*	0.000	0.038	0.341	0.316	0.400	0.184
	*TT*	0.000	0.000	0.049	0.000	0.133	0.027

*DGAT1/Cfr*l	*KK*	0.913	0.077	0.073	0.067	0.158	0.347
	*KA*	0.065	0.769	0.512	0.632	0.600	0.442
	*AA*	0.022	0.154	0.415	0.211	0.333	0.211

N = Nelore; RGxN = Rubia Gallega X Nelore; C = Canchim; B3x = Brangus Three-way cross; BV3x = Braunvieh Three-way cross.

**Table 3:** Least square means and standard errors of the meat traits for each genetic group and subgroup.

		Meat traits			
Genetic group	Subgroup	BT (mm)	TL (%)	REA (cm^2^)	SF (kg)
C	F E	3.75 ± 1.96^AB^	0.02 ± 0.009^AB^	62.51 ± 6.58^C^	3.68 ± 0.64^BC^
	M F	3.69 ± 1.55^AB^	0.02 ± 0.014^AB^	79.98 ± 9.46^A^	3.68 ± 0.65^BC^

N	M A	4.55 ± 0.9^B^	0.01 ± 0.003^AB^	64.90 ± 4.99^C^	4.55 ± 1.28^B^
	M B	4.60 ± 1.42^B^	0.01 ± 0.003^AB^	70.64 ± 8.29^BC^	5.59 ± 1.03^A^
	M D	3.66 ± 1.50^AB^	0.02 ± 0.011^A^	65.33 ± 7.18^C^	3.77 ± 0.62^BC^

RGxN	F C	2.69 ± 1.31^AB^	0.01 ± 0.005^B^	61.69 ± 9.59^C^	3.57 ± 0.73^BC^
	M C	2.25 ± 0.63^A^	0.01 ± 0.004^AB^	62.90 ± 7.56^C^	3.83 ± 1.27^BC^

B3x	M F	4.13 ± 1.44^B^	0.02 ± 0.015^AB^	75.45 ± 7.29^AB^	2.98 ± 0.40^C^

BV3x	M F	3.83 ± 1.31^AB^	0.02 ± 0.012^AB^	74.65 ± 3.16^AB^	3.21 ± 0.46^C^

BT = backfat thickness; TL = total lipids; REA = ribeye area, SF = shear force; N = Nelore; RGxN = Rubia Gallega X Nelore; C = Canchim; B3x = Brangus Three-way cross; BV3x = Braunvieh Three-way cross. The subgroups relate to contemporary criteria. F = female, M = male. A,..., F: slaughter days. ^A, B ,C^ = Differences among genetic subgroups within each meat trait (p < 0.05).

**Table 4:** Least square means and standard errors of the meat traits for genotypes *LEP/Kpn*2I, *TG/Psu*I and *DGAT1/Cfr*l

		Meat traits			
Locus	Genotype	BT (cm)	TL	REA (cm^2^)	SF (kg)
*LEP/Kpn*2I	*CC*	3.42 ± 1.50	0.016 ± 0.012	67.61 ± 9.72	3.68 ± 0.87
	*CT*	3.96 ± 1.60	0.015 ± 0.010	69.20 ± 9.09	3.70 ± 0.88

*TG/Psu*I	*CC*	3.85 ± 1.55	0.021 ± 0.011	73.50 ± 10.25	3.41 ± 0.53
	*CT*	4.00 ± 1.63	0.017 ± 0.013	73.05 ± 8.25	3.30 ± 0.79

*DGAT1/Cfr*l	*AA*	3.19 ± 1.76	0.017 ± 0.012	69.76 ± 10.39	3.64 ± 0.75
	*AK*	3.43 ± 1.46	0.016 ± 0.012	70.19 ± 10.69	3.42 ± 0.72

BT = backfat thickness; TL = total lipids; REA = ribeye area, SF = shear force.
